# Intrauterine Growth Retardation Increases the Susceptibility of Pigs to High-Fat Diet-Induced Mitochondrial Dysfunction in Skeletal Muscle

**DOI:** 10.1371/journal.pone.0034835

**Published:** 2012-04-16

**Authors:** Jingbo Liu, Daiwen Chen, Ying Yao, Bing Yu, Xiangbing Mao, Jun He, Zhiqing Huang, Ping Zheng

**Affiliations:** 1 Institute of Animal Nutrition, Sichuan Agricultural University, Ya'an, Sichuan, People's Republic of China; 2 Key Laboratory of Animal Disease-Resistance Nutrition, Ministry of Education, People's Republic of China; Université Joseph Fourier, France

## Abstract

It has been recognized that there is a relationship between prenatal growth restriction and the development of metabolic-related diseases in later life, a process involved in mitochondrial dysfunction. In addition, intrauterine growth retardation (IUGR) increases the susceptibility of offspring to high-fat (HF) diet-induced metabolic syndrome. Recent findings suggested that HF feeding decreased mitochondrial oxidative capacity and impaired mitochondrial function in skeletal muscle. Therefore, we hypothesized that the long-term consequences of IUGR on mitochondrial biogenesis and function make the offspring more susceptible to HF diet-induced mitochondrial dysfunction. Normal birth weight (NBW), and IUGR pigs were allotted to control or HF diet in a completely randomized design, individually. After 4 weeks of feeding, growth performance and molecular pathways related to mitochondrial function were determined. The results showed that IUGR decreased growth performance and plasma insulin concentrations. In offspring fed a HF diet, IUGR was associated with enhanced plasma leptin levels, increased concentrations of triglyceride and malondialdehyde (MDA), and reduced glycogen and ATP contents in skeletal muscle. High fat diet-fed IUGR offspring exhibited decreased activities of lactate dehydrogenase (LDH) and glucose-6-phosphate dehydrogenase (G6PD). These alterations in metabolic traits of IUGR pigs were accompanied by impaired mitochondrial respiration function, reduced mitochondrial DNA (mtDNA) contents, and down-regulated mRNA expression levels of genes responsible for mitochondrial biogenesis and function. In conclusion, our results suggest that IUGR make the offspring more susceptible to HF diet-induced mitochondrial dysfunction.

## Introduction

Intrauterine growth retardation is a condition that the fetus does not reach its growth potential during gestation period [1]. The pigs, as multifetal domestic animals, exhibit the most severe, naturally occurring IUGR due to the uteroplacental insufficiency [2]. There are increasing evidences shown that IUGR may also have long-term consequences on the offspring [3]. Epidemiological studies have revealed that IUGR is associated with increased risk of metabolic dysfunctions like obesity, diabetes, and abnormal lipid metabolism in adulthood [4], [5]. The long-term influence of abnormal growth during pregnancy on development of the offspring termed metabolic programming, a phenomenon first described by Hales and Barker [6].

Mitochondria are the major sites of reactive oxygen species (ROS) and ATP production in the body [7]. Considering the central role of mitochondria in metabolic regulation, previous studies suggested that mitochondrial dysfunction is an underlying mechanism responsible for the persistent influence, induced by IUGR, on the offspring, which has been observed in offspring subjected to maternal protein restriction, a well established IUGR model [8]–[11]. Likewise, decreased mtDNA content, impaired oxidative phosphorylation process, reduced mitochondrial enzymes activities, and down-regulated mRNA expression levels of mitochondrial encoded genes were observed in the liver and skeletal muscle of bilateral uterine artery ligation induced IUGR offspring [12]–[14]. Using microarray technology, abnormal expression patterns of several mitochondrial function-related genes were also determined in IUGR offspring [10], [15]. It has been suggested that the effect of intrauterine experience such as prenatal undernutrition on later metabolic function has an adaptive origin [16], in that they are a result of evolved processes designed to maintain growth fitness across a range of potential environments. Developmental plasticity increases the adaptivity of the fetal in postnatal life when the experienced environment matches the expectation [17]. However, mismatch between postnatal environment and predicted surrounding aggravates the metabolic abnormalities in the offspring [18]. Previous study has also demonstrated that fetal origins of hyperphagia, obesity, and hypertension postnatal were amplified by hypercaloric nutrition during postnatal life [19].

The mechanisms behind this phenomenon remain poorly understood. Because of its similar homology to human, the pigs have been recognized as one of the ideal models for the study of clinic nutrition [20]. Studying the effect of high fat diet on mitochondrial function in skeletal muscle of pigs may provide information on the variability in diet-induced metabolic responses between normal birth weight (NBW) and IUGR offspring. Based on the finding that high fat diet impaired mitochondrial function [21], [22], we hypothesize that feeding a high fat diet to the IUGR offspring increases the susceptibility of pigs to mitochondrial dysfunction in skeletal muscle. Therefore, the aim of this study was to assess the difference in metabolic responses, mitochondrial DNA biogenesis, and mitochondrial function between NBW and IUGR pigs in response to high fat diet.

## Materials and Methods

### Ethics Statement

The experimental procedures were approved by the University of Sichuan Agricultural Animal Care Advisory committee, and followed the current law of animal protection (NRC 1996) [23].

### Animal models and diets

Normal birth weight and IUGR piglets could be defined following the criteria we described previously [24]. The average birth weight of NBW and IUGR piglets used in the present study were 1.49 kg and 0.97 kg, respectively. A total of sixteen NBW and sixteen IUGR male pigs at 5 month old were selected and allotted to control diet (C) and high fat diet (HF) groups (average body weight of NBW and IUGR were 89.1 kg and 77.3 kg, respectively). This produced 4 experimental groups (birth weight/diet); NBW/C, NBW/HF, IUGR/C, and IUGR/HF (n = 8 per group). The diets (Table 1) were formulated to meet or exceed the nutrient requirements of growing-finishing pigs [25]. The cornstarch and lard were purchased from Chengdu food market (Chengdu, Sichuan, China). The C and HF diets were different in fat and carbohydrate content. There were no discrepancies for other nutrient components.

**Table 1:** Composition of experimental diet (as fed-basis).

Ingredients (% of diet)	Control diet	High fat diet
Corn	65.00	65.00
Corn starch	10.00	–
Soybean meal	16.50	16.50
Lard	–	10.00
Wheat bran	5.00	5.00
Limestone	0.80	0.80
Dicalcium phosphate	1.58	1.58
Sodium chloride	0.50	0.50
Trace mineral Premix^a^	0.30	0.30
Vitamin Premix^b^	0.10	0.10
Choline chloride (50%)	0.10	0.10
L-Lys	0.12	0.12
Total	100.00	100.00

a Supplied (per kg diet): Fe as FeSO_4_.7H_2_O, 160 mg; Mn as MnSO_4_.7H_2_O, 30 mg; Zn as Zn SO_4_, 160 mg; Cu as CuSO_4_.5H_2_O, 30 mg; Se as NaSeO_3_, 0.5 mg; and I as KI, 0.6 mg.

b Supplied (per kg diet): 12,800 IU vitamin A, 44 IU vitamin E, 2,600 IU vitamin D, 4 mg vitamin K, 2.4 mg vitamin B_1_, 8.8 mg riboflavin, 32 mg niacin, 4 mg pantothenic acid, 0.5 mg biotin, 2 mg folic acid, and 0.05 mg vitamin B_12_.

### Animal housing and tissue sampling

Pigs were housed individually in metabolic cages with woven wire flooring and were given ad libitum access to water through a water nipple. During the 28 d experimental period, pigs were fed their assigned diets (C or HF) three times per day (7:00, 12:00, and 18:00) to ensure that all pigs received an ad libitum access to diet. Body weight and feed intake of pigs were recorded weekly throughout the trial. At the end of the experiment, blood samples were collected by venipuncture at 8:00 and stored at −20°C. All pigs were sacrificed as we previously described [26], and the semitendinosus were collected and stored at −80°C for further analysis.

### Biochemical measurements

Plasma glucose and triglyceride concentrations were measured using a Konelab 20 autoanalyzer. Leptin concentration in plasma was measured with commercial RIA kits purchased from Beijing North Institute of Biotechnology (Beijing, China). Insulin was measured with the use of electro-chemiluminescence immunoassays (Roche Diagnostics, Meylan, France). The intra- and inter-assay coefficients of variations were 5% and 10%, respectively. The concentrations of triglyceride, lactate, adenosine triphosphate (ATP), MDA, glycogen, and protein carbonyls in skeletal muscle were determined using colorimetric methods with spectrophotometer (Nanjing Jiancheng Institute of Bioengineering, Jiangsu, China) according to the instructions of the kits. The activities of citrate synthase (CS), lactate dehydrogenase (LDH), aconitase, superoxide dismutase (SOD), malic enzyme (ME) and glucose-6-phosphate dehydrogenase (G6PD) in skeletal muscle were assayed according to the methods described elsewhere [27]–[29]. F_0_F_1_ATPase activity was determined as ATPase after ATP hydrolysis with an ATP-regenerating system coupled to NADPH oxidation [30].

### Mitochondrial oxygraphic and membrane potential measurements

Fresh muscle samples were rapidly treated for mitochondrial isolation as reported previously [31]. Lowry micromethod kit (Sigma-Aldrich) was selected to determine mitochondrial protein concentration. Processed mitochondria were used for oxygen consumption assay at 37°C in a thermostatically controlled oxygraph apparatus equipped with a Clark electrode (Hansatech Instruments Ltd., Norfolk, UK). State 4 respiration rates were measured by the addition of 10 mM glutamate/5 mM malate, or 5 mM succinate in the presence of 2 µM rotenone. State 3 respiration was induced by addition of 100 µM ADP. Oxygen consumption of isolated skeletal muscle mitochondria in state 3 and state 4 and the respiratory control index (RCI) were measured as reported previously [31]. Mitochondria membrane potential (ΔΨ) was determined at 37°C in the presence of 5 mM glutamate plus 5 mM malate or 5 mM succinate plus 2 µM rotenone and 5 µM oligomycin by a Clarke and a tetraphenylphosphonium electrode (WPI, Berlin, Germany). Membrane potential was calculated by a modified Nernst equation [31].

### DNA and RNA extraction

Total DNA was extracted from the skeletal muscle of each piglet using a DNAiso Reagent (TaKaRa, Dalian, China). Total RNA was extracted using Trizol Reagent (TaKaRa, Dalian, China) and further purified by Qiagen RNeasy Mini kit (Qiagen, Valencia, CA, USA). All the procedures were according to the manufacturer's protocol. RNA concentration was determined using spectrophotometry based on absorbance at 260 nm and integrity was monitored using the Agilent 2100 Bioanalyzer (Agilent Technologycies, USA).

### Quantitative Real-Time PCR

The content of mtDNA relative to nuclear genomic DNA was measured by coamplifying the mt D-loop and the nuclear-encoded β-actin gene using real-time PCR assay. The amount of mt D-loop and β-actin gene were quantified by fluorescent probes. The sequence of primers and probes were shown in Table 2. PCR amplification was carried out in a 20-µL reaction volume consisting of 8 µL TaqMan Universal Master mix, 1 µL enhance solution, 1 µL each of forward and reverse primers, 1 µL probes, 7 µL ddH_2_O and 1 µL DNA. The cycling conditions were as follows, 95°C for 10 s, 50 cycles involving a combination of 95°C for 5 s and 60°C for 25 s, and 95°C for 10 s. Each sample was amplified in triplicate. The fluorescence spectra were monitored by CFX-96 Real-Time PCR detection System (Bio-Rad, USA). The ratio of mtDNA to genomic DNA content was calculated as ΔCt (mt ΔCt _D-loop_ – nuclear Ct _β-actin_). The relative expression (RE) indicates the factorial difference in mtDNA content between each group. RE was calculated as 2^−ΔΔCt^, where ΔΔCt = ΔCt_mtDNA content in other group_ – ΔCt _mtDNA content in the control_.

**Table 2:** Primer and probe sequences used for determination of mtDNA content.

Gene name		Sequence	Accession number	Product size (bp)
Mitochondrial D-loop (mt D-loop)	F	5′- GATCGTACATAGCACATATCATGTC-3′	AF276923	198
	R	5′-GGTCCTGAAGTAAGAACCAGATG -3′		
	P	5′-(FAM) CCAGTCAACATGCGTATCACCACCA (Eclipse)-3′		
β-actin	F	5′-CCCCTCCTCTCTTGCCTCTC -3′	DQ452569	74
	R	5′-AAAAGTCCTAGGAAAATGGCAGAAG -3′		
	P	5′-(FAM) TGCCACGCCCTTTCTCACTTGTTCT (Eclipse)-3′		

Expression levels of target gene in the liver were analysed by real-time PCR using SYBR Premix Ex Taq reagents (TaKaRa, Dalian, China) and CFX-96 Real-Time PCR detection System (Bio-Rad, USA). mRNA was reverse-transcribed using PrimeScript™ reagent kit (TaKaRa, Dalian, China) according to manufacturer's instructions. The PCR system consisted of 5 µL SYBR Premix Ex Taq™ (2×), 1 µL forward primers, 1 µL reverse primers, 2 µL ddH_2_O and 1 µL cDNA in a total volume of 10 µL. The primers are presented in Table 3. Cycling conditions were as follows: a pre-run at 95°C for 10 s, 40 cycles of denaturation step at 95°C for 5 s, followed by a 60°C annealing step for 25 s and a 72°C extension step for 15 s. Melting curve conditions were: 1 cycle of denaturation at 95°C for 10 s, then 65°C change to 95°C with temperature change velocity at 0.5°C/s. The β-actin gene was used as the reference gene to normalize mRNA expressions of target genes. Gene expression data from replicate samples were analyzed using the Pfaffl method to between the cassava and maize cycle threshold values [32].

**Table 3:** Primer sequences of target genes used for real-time RT-PCR.

Gene^a^	5′-Primer (F)	3′-Primer (R)	Accession number	Product size (bp)
PGC-1α	CCCGAAACAGTAGCAGAGACAAG	CTGGGGTCAGAGGAAGAGATAAAG	NM_213963	111
TFAM	GGTCCATCACAGGTAAAGCTGAA	ATAAGATCGTTTCGCCCAACTTC	AY923074.1	167
NRF-1	GCCAGTGAGATGAAGAGAAACG	CTACAGCAGGGACCAAAGTTCAC	AK237171.1	166
mt SSB	CTTTGAGGTAGTGCTGTGTCG	CTCACCCCTGACGATGAAGAC	AK352341.1	143
mt polr	CTTTGAGGTTTTCCAGCAGCAG	GCTCCCAGTTTTGGTTGACAG	XM_001927064.1	119
SIRT-1	TGACTGTGAAGCTGTACGAGGAG	TGGCTCTATGAAACTGCTCTGG	EU030283.2	143
CcOX I	ATTATCCTGACGCATACACAGCA	GCAGATACTTCTCGTTTTGATGC	AJ950517.1	127
CcOX IV	CCAAGTGGGACTACGACAAGAAC	CCTGCTCGTTTATTAGCACTGG	AK233334.1	131
CcOX V	ATCTGGAGGTGGTGTTCCTACTG	GTTGGTGATGGAGGGGACTAAA	AY786556.1	160
Cyt c	TAGAAAAGGGAGGCAAACACAAG	GGATTCTCCAGGTACTCCATCAG	NM_001129970.1	154
ATPS	TGTCCTCCTCCCTATCACACATT	TAGTGGTTATGACGTTGGCTTGA	AK230503	116
ND4	TTATTGGTGCCGGAGGTACTG	CCCAGTTTATTCCAGGGTTCTG	NM_001097468	112
Glucokinase	CTTTTCCCTCCCACACTGCTAT	GACTCCTCTTCCTGAGACCCTCT	AK233298.1	119
CS	CCTTTCAGACCCCTACTTGTCCT	CACATCTTTGCCGACTTCCTTC	M21197.1	127
β-actin	TCTGGCACCACACCTTCT	TGATCTGGGTCATCTTCTCAC	DQ178122	114

a Gene abbreviations: PGC-1α, PPARγ coactivator-1α; TFAM, mitochondrial transcription factor A; NRF-1, nuclear respiratory factor-1; mt SSB, mitochondrial single-strand DNA-binding protein; mt polr, mitochondrial polymerase r; SIRT-1, mammalian silencing information regulator; CcOX, cytochrome c oxidase; Cyt c, cytochrome c; ATPS, ATP synthase; ND4, NADPH dehydrogenase 4; CS, citrate synthase.

### Statistical analyses

Statistical analyses were carried out using SAS statistical packages (SAS Institute, Cary, NC, USA). Comparisons between two groups were evaluated using an unpaired *t* test. Differences between groups were determined by two-way ANOVA with birth weight and diet as factors; *P*<0.05 was considered statistical significant, post-hoc analyses were performed. Data are presented as mean ± SEM.

## Results

### Growth performance, metabolites and hormones

**Figure 1:**
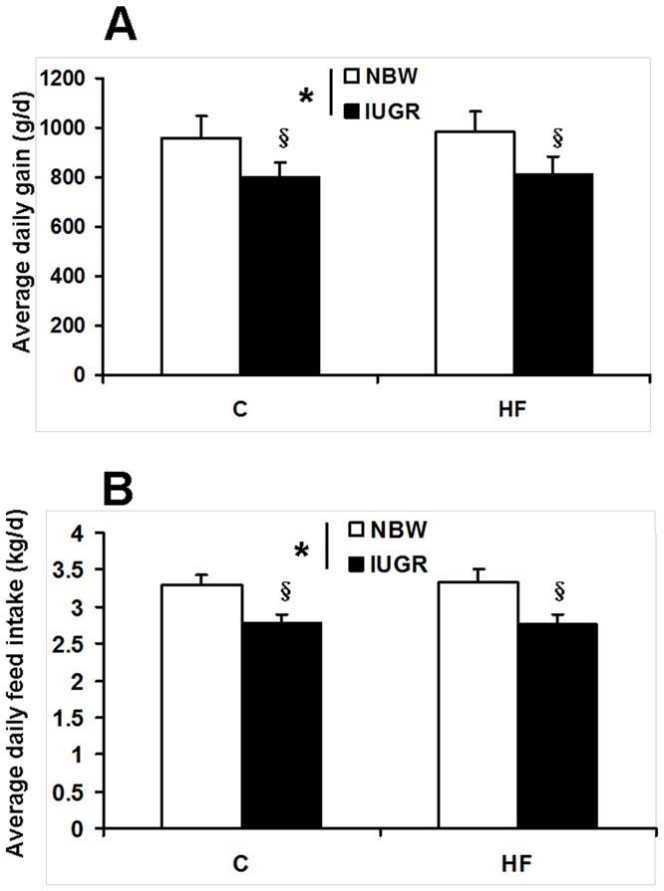
Influence of IUGR and diet on average daily gain (A) and feed intake (B) of pigs. C: Control diet; HF: High-fat diet; NBW: Normal birth weight; IUGR: Intrauterine growth retardation. Data are expressed as group means ± SEM. *P values next to each label represent the significance in the effect for each source of variation (IUGR or diet) as calculated by ANOVA. ^§^ ***P***<0.05 in post hoc test comparing IUGR and NBW offspring receiving the same diet (n = 8 by group).

**Figure 2:**
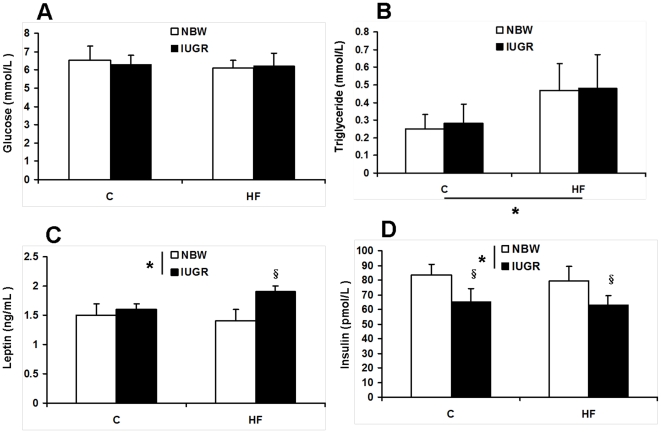
Influence of IUGR and diet on plasma concentrations of glucose (A), triglyceride (B), leptin (C) and insulin (D). C: Control diet; HF: High-fat diet; NBW: Normal birth weight; IUGR: Intrauterine growth retardation. Data are expressed as group means ± SEM. *P values next to each label represent the significance in the effect for each source of variation (IUGR or diet) as calculated by ANOVA. ^§^ ***P***<0.05 in post hoc test comparing IUGR and NBW offspring receiving the same diet (n = 8 by group).

**Figure 3:**
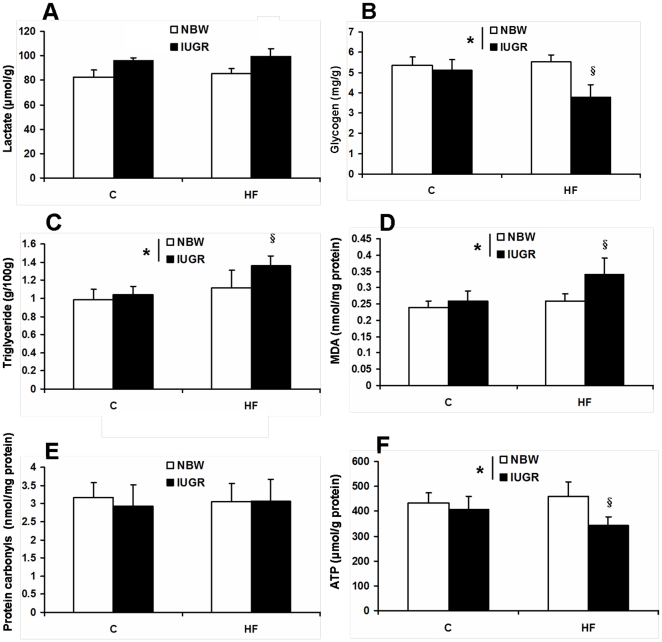
Influence of IUGR and diet on concentrations of lactate (A), glycogen (B), triglyceride (C), MDA (D), protein carbonyls (E) and ATP (F) in skeletal muscle of pigs. C: Control diet; HF: High-fat diet; NBW: Normal birth weight; IUGR: Intrauterine growth retardation. Data are expressed as group means ± SEM. *P values next to each label represent the significance in the effect for each source of variation (IUGR or diet) as calculated by ANOVA. ^§^ ***P***<0.05 in post hoc test comparing IUGR and NBW offspring receiving the same diet (n = 8 by group).

We found IUGR significantly reduced the average daily gain and feed intake of the offspring (*P*<0.05, Figure 1A and 1B). HF diet has no effect on the average daily gain and feed intake during the 28-d treatment period (*P*>0.05, Figure 1A and 1B). There was no significant difference in plasma glucose concentration among the four groups (*P*<0.05, Figure 2A). Feeding HF diet to pigs increased plasma triglyceride content (*P*<0.05, Figure 2B). Plasma insulin concentration was decreased in IUGR piglets (*P*<0.05, Figure 2D). Moreover, leptin content in plasma of IUGR pigs was increased when fed a HF diet (*P*<0.05, Figure 2C). We also determined the concentrations of several metabolites to assess the mitochondrial function in skeletal muscle (Figure 3). Intrauterine growth retardation reduced both glycogen and ATP concentrations in skeletal muscle of pigs receiving HF diet was observed in the present study (*P*<0.05, Figure 3B and 3F). However, skeletal muscle triglyceride and MDA contents were elevated in IUGR pigs fed a HF diet (*P*<0.05, Figure 3C and 3D). Skeletal muscle lactate content and protein carbonyls concentrations were not affected by birth weight and HF diet (*P*>0.05, Figure 3A and Figure 3E).

### Enzyme activities in skeletal muscle

**Figure 4:**
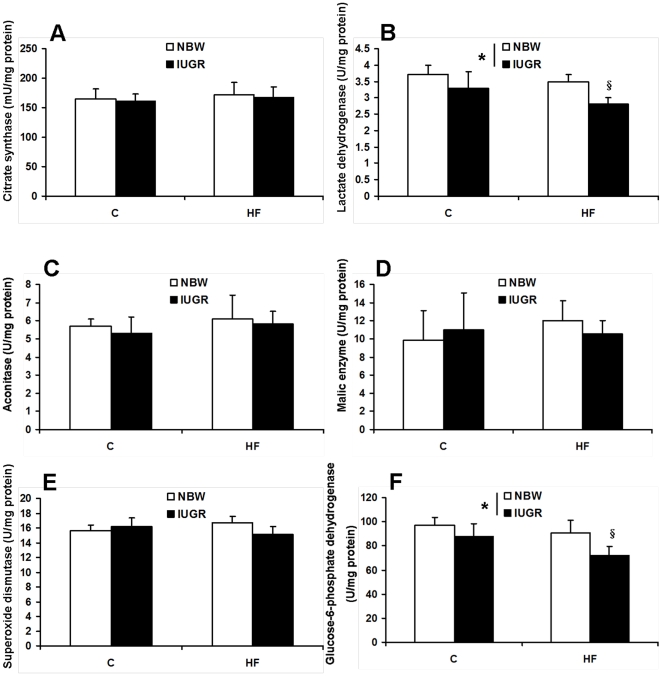
Influence of IUGR and diet on activities of CS (A), LDH (B), aconitase (C), ME (D), SOD (E) and G6PD (F) in skeletal muscle of pigs. C: Control diet; HF: High-fat diet; NBW: Normal birth weight; IUGR: Intrauterine growth retardation. Data are expressed as group means ± SEM. *P values next to each label represent the significance in the effect for each source of variation (IUGR or diet) as calculated by ANOVA. ^§^ ***P***<0.05 in post hoc test comparing IUGR and NBW offspring receiving the same diet (n = 8 by group).

**Figure 5:**
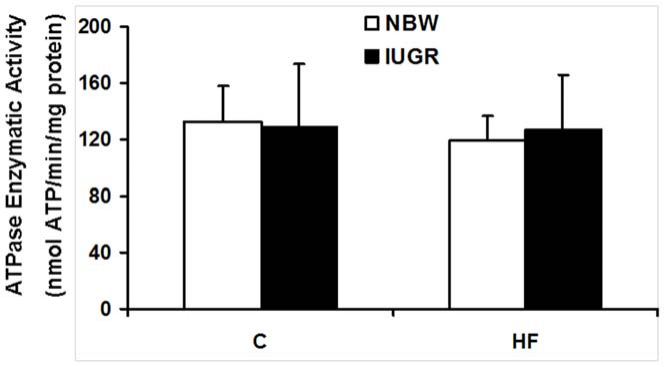
Influence of IUGR and diet on enzymatic activity of mitochondrial F_0_F_1_-ATPase in skeletal muscle of pigs. C: Control diet; HF: High-fat diet; NBW: Normal birth weight; IUGR: Intrauterine growth retardation. Data are expressed as group means ± SEM. *P values next to each label represent the significance in the effect for each source of variation (IUGR or diet) as calculated by ANOVA. ^§^ ***P***<0.05 in post hoc test comparing IUGR and NBW offspring receiving the same diet (n = 8 by group).

The activities of several key enzymes involved in energy metabolism and antioxidant function were measured. Our results showed that the activities of CS, aconitase, ME, and SOD were not affected by birth weight and HF diet (*P*>0.05, Figure 4). The activities of LDH and G6PD were both significantly decreased in skeletal muscle of IUGR piglets fed a HF diet (*P*<0.05, Figure 4B and 4F). F_0_F_1_ATPase activity was not affected by diet and birth weight (*P*>0.05, Figure 5).

### Mitochondrial respiratory chain activity and membrane potential

**Figure 6:**
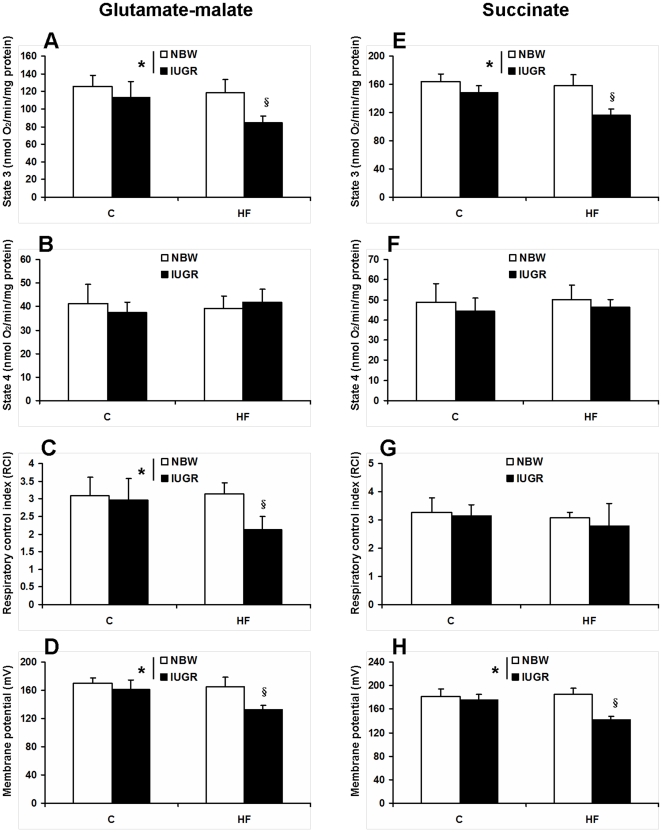
Influence of IUGR and diet on mitochondrial respiratory activities and membrane potential in skeletal muscle of pigs. Glutamate-malate or succinate was used as the oxidative substrate. C: Control diet; HF: High-fat diet; NBW: Normal birth weight; IUGR: Intrauterine growth retardation. Data are expressed as group means ± SEM. *P values next to each label represent the significance in the effect for each source of variation (IUGR or diet) as calculated by ANOVA. ^§^ ***P***<0.05 in post hoc test comparing IUGR and NBW offspring receiving the same diet (n = 8 by group).

Oxidative phosphorylation capacity was determined by measuring oxygen consumption in the presence of an oxidative substrate (glutamate-malate or succinate). State 3 refers to oxygen consumption stimulated by ADP and state 4 represents the oxygen consumption independent of ADP phosphorylation, whereas the RCI is calculated by the ratio of state 3/state 4. Although muscle mitochondria from IUGR pigs fed the HF diet exhibited a decreased in ADP-stimulated (state 3) respiration (*P*<0.05, Figure 6A and 6E), ADP-independent (state 4) respiration did not differ across treatment groups (*P*>0.05, Figure 6B and 6F). Respiratory control index is reduced in IUGR pigs fed a HF diet when glutamate-malate was used as oxidative substrate (*P*<0.05, Figure 6C). ΔΨ is the important component of proton motive force for ATP synthesis, which is reduced in skeletal muscle of IUGR offspring fed the HF diet (*P*<0.05, Figure 6D and 6H).

### Mitochondrial DNA contents

**Figure 7:**
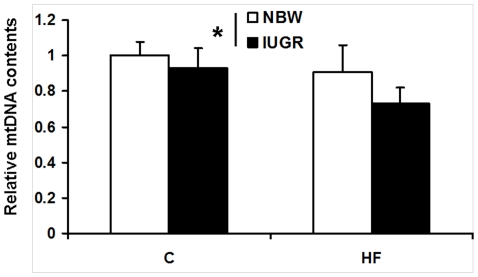
Influence of IUGR and diet on relative mtDNA contents in skeletal muscle of pigs. C: Control diet; HF: High-fat diet; NBW: Normal birth weight; IUGR: Intrauterine growth retardation. Data are expressed as group means ± SEM. *P values next to each label represent the significance in the effect for each source of variation (IUGR or diet) as calculated by ANOVA. ^§^ ***P***<0.05 in post hoc test comparing IUGR and NBW offspring receiving the same diet (n = 8 by group).

In contrast, HF-feeding significantly decreased mitochondrial DNA (mtDNA) contents in skeletal muscle were observed in the present study, especially in IUGR offspring (*P*<0.05, Figure 7).

### Gene mRNA expression levels analysis

#### Factors involved in mtDNA biogenesis: PGC-1α, TFAM, SIRT-1, NRF-1, mt SSB, mt polr mRNA levels

**Figure 8:**
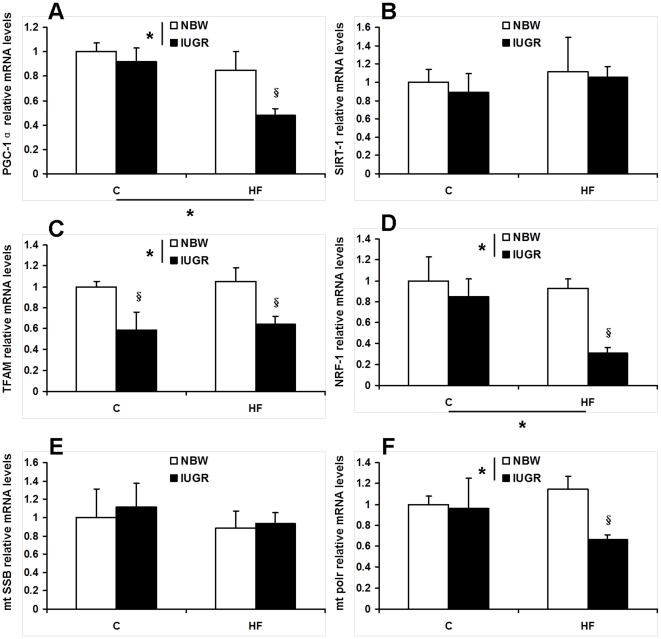
Influence of IUGR and diet on mRNA levels of peroxisomal proliferator-activated receptor-γ coactivator-1α (PGC-1α, A), mitochondrial transcription factor A (TFAM, B), mammalian silencing information regulator-2α (SIRT-1, C), nuclear respiratory factor-1 (NRF-1, D), mitochondrial single-strand DNA-binding protein (mt SSB, E) and mitochondrial polymerase r (mt polr, F) in skeletal muscle of pigs. C: Control diet; HF: High-fat diet; NBW: Normal birth weight; IUGR: Intrauterine growth retardation. Data are expressed as group means ± SEM. *P values next to each label represent the significance in the effect for each source of variation (IUGR or diet) as calculated by ANOVA. ^§^ ***P***<0.05 in post hoc test comparing IUGR and NBW offspring receiving the same diet (n = 8 by group).

We found that peroxisomal proliferator-activated receptor-γ coactivator-1α (PGC-1α) and nuclear respiratory factor-1 (NRF-1) mRNA levels were significantly decreased in skeletal muscle from IUGR pigs (*P*<0.05), with a significant effect of HF diet (*P*<0.05, Figure 8A and 8D). mRNA expression abundance of mammalian silencing information regulator-2α (SIRT-1) and mt single-strand DNA-binding protein (mt SSB) were similar among groups (*P*>0.05, Figure 8B and 8E). Intrauterine growth retardation reduced mRNA expression level of mt transcription factor A (TFAM), independent of diet (*P*<0.05, Figure 8C). The consumption of a HF diet resulted in a significant decrease in mt polymerase r (mt polr) expression level in IUGR pigs (*P*<0.05, Figure 8F).

#### Factors involved in mitochondrial function: Glucokinase, CS, ATPS, CcOX I, CcOX IV, CcOX V, Cyt c, ND4 mRNA levels

**Figure 9:**
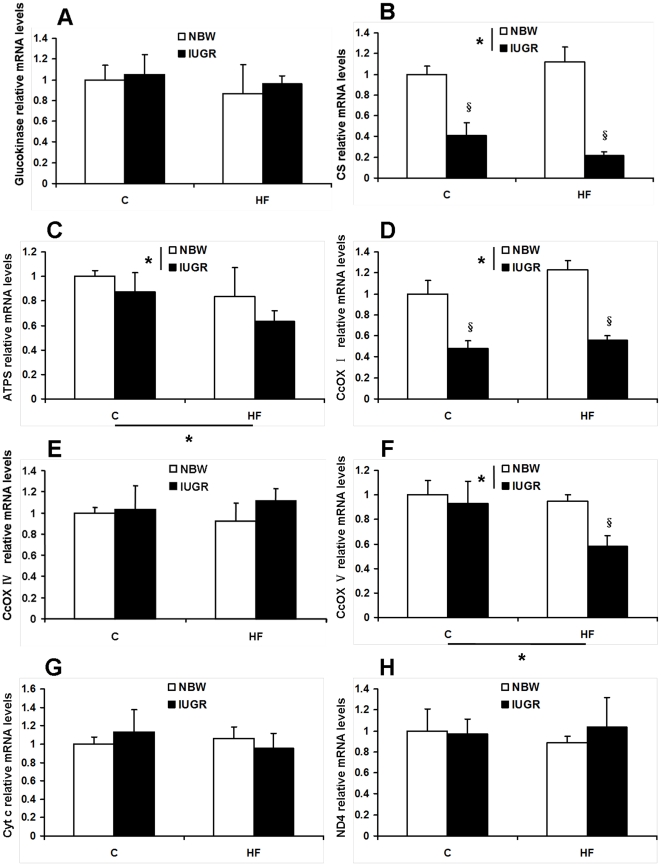
Influence of IUGR and diet on mRNA levels of Glucokinase (A), citrate synthase (CS, B), adenosine triphosphate synthase (ATPS, C), cytochrome c oxidase I (CcOX I, D), cytochrome c oxidase IV (CcOX IV, E), cytochrome c oxidase V (CcOX V, F), Cytochrome c (Cyt c, G) and NADH dehydrogenase subunit 4 (ND4, H) in skeletal muscle of pigs. C: Control diet; HF: High-fat diet; NBW: Normal birth weight; IUGR: Intrauterine growth retardation. Data are expressed as group means ± SEM. *P values next to each label represent the significance in the effect for each source of variation (IUGR or diet) as calculated by ANOVA. ^§^ ***P***<0.05 in post hoc test comparing IUGR and NBW offspring receiving the same diet (n = 8 by group).

There was no significant difference in mRNA expression levels of glucokinase, cytochrome c oxidase IV (CcOX IV), Cytochrome c (Cyt c) and NADH dehydrogenase subunit 4 (ND4) between NBW and IUGR pigs, with no significant effect of diet (*P*>0.05, Figure 9A, 9E, 9G and 9H). In contrast, IUGR reduced mRNA expression levels of citrate synthase (CS) and cytochrome c oxidase I (CcOX I) in skeletal muscle of pigs (*P*<0.05). No effect of HF diet on gene expression levels of CS and CcOX I was observed (*P*>0.05, Figure 9B and 9D). Feeding a HF diet to the offspring resulted in a significant decrease in mRNA levels of adenosine triphosphate synthase (ATPS) and cytochrome c oxidase V (CcOX V, *P*<0.05), especially in those born with IUGR (*P*<0.05, Figure 9C and 9F).

## Discussion

The intriguing findings of the present study were that IUGR induces different changes in skeletal muscle mitochondrial function of pigs in response to HF diet compared with NBW pigs. Specifically, our results demonstrated that IUGR increases the susceptibility to HF diet-induced alterations in lipid metabolism, hormone secretion, mitochondrial respiratory function, antioxidant process, and mRNA levels of genes involved in mitochondrial biogenesis and function. Therefore, as shown by us and others [21], [33], [34], in consumption of a HF diet, IUGR offspring exhibit mitochondrial dysfunction in skeletal muscle, which may provide a potential molecular pathway responsible for the increased risk of developing metabolic syndrome.

Intrauterine growth retardation caused by undernourishment of the mother is known to exhibit an exacerbated weight gain after birth in experimental animals [35]. Contrary to the results from rats, we found a significant decrease in growth performance and feed intake of IUGR offspring compared with NBW offspring independent of the diet they were receiving in the present study. This is in agreement with previous studies in pigs, which observed that low birth weight pigs is associated with reduced average daily gain and body weight in adult life [36], [37]. Taken together, we suggest that different effect of IUGR on postnatal growth performance of offspring is connected to the methods used to induce IUGR.

Previous studies in rats demonstrated that both IUGR and HF diet were key factors to influence insulin resistance and lipid metabolism [19], [38]–[40]. In the current study, we observed there is an interaction between IUGR and HF diet by showing that IUGR offspring exhibited variability in circulating levels of leptin when fed a C or HF diet. Furthermore, previous study also reported that IUGR is associated with impairment in glucose homeostasis and lipid profile in adult life [41]. However, we found no difference in glucose homeostasis between NBW and IUGR offspring. It is possible that 6 month is too short for pigs to detect changes in circulating glucose levels. The abnormal insulin sensitivity of IUGR offspring was observed in the present study evidenced by decreased plasma insulin levels, which was consistent with previous studies in pigs and rats [37], [42]. In this study, HF-fed IUGR pigs have higher muscle triglyceride content. However, no alteration was found in IUGR offspring when fed the C diet. Previous studies found IUGR reduced or had no effects on muscle lipid content in pigs [43], [44]. Therefore, taken together these results, we suggest that fat content and energy level of the diet are important factors for regulating lipid deposition in skeletal muscle of IUGR pig.

Mitochondrial dysfunction was an important event in the development of insulin resistance and metabolic disorders in adult life [45]. Mitochondria play a central role in generating reactive oxygen species (ROS) and providing energy for cellular needs through producing ATP [46]. Impairment of mitochondrial function leads to destruction of oxidative phosphorylation and antioxidant system, and reduction in the synthesis of ATP and glycogen [13]. In the present study, we observed IUGR reduced state 3 respiration and RCI, decreased concentration of ATP and glycogen, and increased MDA production in skeletal muscle of pigs when exposed to a HF diet. It is well established that maternal malnutrition-induced IUGR decreased ATP production through impaired oxidative phosphorylation process and enhanced oxidative stress in liver and skeletal muscle of the offspring [13], [47]. However, the changes in concentrations of ATP and glycogen were not exhibited in IUGR offspring when fed the C diet in this trial. Therefore, we suggest that dietary factor was an effective regulator in terms of triggering the harmful effect of IUGR on mitochondrial function, and such a defect in mitochondria leads to reduction in the production of ATP available from oxidative phosphorylation. Mitochondrial membrane potential is the major component of proton motive force used for ATP synthesis. HF-diet induced a significant reduction in the ΔΨ in IUGR pigs, which can contribute to the decreased ATP content in skeletal muscle of IUGR offspring. Intrauterine growth retardation changes the concentration of skeletal muscle ATP without any effect on the activity of ATPase. Such results may be caused by the mitochondrial proton leak, which alters the efficiency of oxidative phosphorylation activity [48]. Both G6PD and LDH play an important role in energy metabolism. Consistent with a previous study [42], we observed a decrease in G6PD and LDH activities in skeletal muscle of IUGR offspring fed the HF diet compared with NBW pigs. One possible mechanism responsible for this phenomenon is mitochondrial dysfunction in skeletal muscle of IUGR, which has been reported previously [9]–[11].

Previous investigation strongly suggested that quantitative abnormality of mtDNA was associated with the risk of metabolic symptom [49]. In the IUGR rats, mtDNA content was found to be reduced in liver and skeletal muscle of the offspring [8]. Similar to previous studies, skeletal muscle mtDNA level was decreased in IUGR pigs when fed the HF diet in this trial. However, as CS activity can be considered a measure of mitochondrial mass [50], our results found no difference in activity of CS between IUGR and NBW pigs. The discrepancies between mtDNA content and CS activity among groups revealed that CS activity may be also impacted by other factors.

Mitochondrial DNA content is under the control of mitochondrial biogenesis, which needs the interaction of multiple transcriptional factors [51]. PGC-1α and NRF-1, transcriptional coactivators of nuclear receptors to modulate mitochondrial biogenesis, were down-regulated in IUGR pigs fed the HF diet in our study. Expression level of TFAM, a nuclear-encoded regulator of mtDNA replication and transcription that could be regulated by PGC-1a and NRF-1 to initiate mitochondrial biogenesis [52], [53], was also decreased in skeletal muscle of IUGR piglets. Furthermore, mtDNA replication and repair were affected by mt SSB and mt polr [54]. Expression level of mt polr was reduced in IUGR offspring, whereas mRNA level of mt SSB did not differ among the groups. Consistent with other reports in rats [8], [55], abnormal expression patterns of genes responsible for mtDNA biogenesis in skeletal muscle of IUGR pigs were also observed, especially for the offspring fed the HF diet. The changes in mRNA expression of genes involved in mitochondrial biogenesis may account for the differences in mtDNA content.

Mitochondrial dysfunction can lead to changes in glycolysis, oxidative phosphorylation, TCA cycle, and ATP production [56]. The reduction of ATP concentration in skeletal muscle of IUGR offspring reflected that these processes might be impaired. The determination of mRNA expression abundance of genes that have been described to play a central role in the process of oxidative phosphorylation, TCA cycle and ATP formation may provide some insights on the mitochondrial function. In the present study, as expected, mRNA levels of several genes responsible for mitochondrial function were down-regulated in skeletal muscle of HF-fed IUGR pigs. In agreement with previous studies in rats [8], [57], we found that IUGR decreased CS, CcOX I and CcOX V mRNA expression level in skeletal muscle. However, there is no evidence of any impact of HF or birth weight on mRNA expression abundance of glucokinase, CcOX IV, Cyt c and ND4 in our animal model. ATPS is known to play an important role in regulation of ATP formation, and its mRNA expression abundance is often considered as a good indicator of ATP concentration in tissues. Therefore, the decreased mRNA level of ATPS may contribute to the changes in skeletal muscle ATP content of offspring in this trial.

In conclusion, our study indicates that IUGR increases the susceptibility of pigs to HF diet-induced mitochondrial dysfunction. Moreover, a previous study also suggested that IUGR leads to the offspring preference for fat as energy source, resulting in mitochondrial dysfunction and subsequent abnormal metabolic process [15]. Further investigations are warranted to determine whether IUGR pigs fed such a HF diet during early period of life will have persistent impact on mitochondrial function.

## References

[1] Scifres CM, Nelson DM (2009). Intrauterine growth restriction, human placental development and trophoblast cell death.. J Physiol.

[2] Wu G, Bazer FW, Wallace JM, Spencer TE (2006). Board-invited review: intrauterine growth retardation: implications for the animal sciences.. J Anim Sci.

[3] Godfrey K, Cameron I, Hanson M (2006). Long-term consequences of foetal restriction.. Current Obstetrics & Gynaecology.

[4] Barker DJ (2000). In utero programming of cardiovascular disease.. Theriogenology.

[5] Godfrey KM, Barker DJ (2000). Fetal nutrition and adult disease.. Am J Clin Nutr.

[6] Hales CN, Barker DJ (1992). Type 2 (non-insulin-dependent) diabetes mellitus: the thrifty phenotype hypothesis.. Diabetologia.

[7] Hock MB, Kralli A (2009). Transcriptional control of mitochondrial biogenesis and function.. Annu Rev Physio.

[8] Park KS, Kim SK, Kim MS, Cho EY, Lee JH (2003). Fetal and early postnatal protein malnutrition cause long-term changes in rat liver and muscle mitochondria.. J Nutr.

[9] Mortensen OH, Olsen HL, Frandsen L, Nielsen PE, Nielsen FC (2010). Gestational protein restriction in mice has pronounced effects on gene expression in newborn offspring's liver and skeletal muscle; protective effect of taurine.. Pediatr Res.

[10] Mortensen OH, Olsen HL, Frandsen L, Nielsen PE, Nielsen FC (2010). A maternal low protein diet has pronounced effects on mitochondrial gene expression in offspring liver and skeletal muscle; protective effect of taurine.. J Biomed Sci.

[11] Lee YY, Park KS, Pak YK, Lee HK (2005). The role of mitochondrial DNA in the development of type 2 diabetes caused by fetal malnutrition.. J Nutr Biochem.

[12] Peterside IE, Selak MA, Simmons RA (2003). Impaired oxidative phosphorylation in hepatic mitochondria in growth-retarded rats.. Am J Physiol Endocrinol Metab.

[13] Selak MA, Storey BT, Peterside I, Simmons RA (2003). Impaired oxidative phosphorylation in skeletal muscle of intrauterine growth-retarded rats.. Am J Physiol Endocrinol Metab.

[14] Lane RH, Flozak AS, Oqata ES, Bell GI, Simmons RA (1996). Altered hepatic gene expression of enzymes involved in energy metabolism in the growth-retarded fetal rat.. Pediatr Res.

[15] Morris TJ, Vickers M, Gluckman P, Gilmour S, Affara N (2009). Transcriptional profiling of rats subjected to gestational undernourishment: implications for the developmental variations in metabolic traits.. PLoS One.

[16] Gluckman PD, Hanson MA, Beedle AS (2007). Early life events and their consequences for later disease: a life history and evolutionary perspective.. Am J Hum Biol.

[17] Gluckman PD, Hanson MA, Cooper C, Thornburg KL (2008). Effect of in utero and early-life conditions on adult health and disease.. N Engl J Med.

[18] Godfrey KM, Lillycrop KA, Burdge GC, Gluckman PD, Hanson MA (2007). Epigenetic mechanisms and the mismatch concept of the developmental origins of health and disease.. Pediatr Res.

[19] Vickers MH, Breier BH, Cutfield WS, Hofman PL, Gluckman PD (2000). Fetal origins of hyperphagia, obesity, and hypertension and postnatal amplification by hypercaloric nutrition.. Am J Physiol Endocrinol Metab.

[20] Merrifield CA, Lewis M, Claus SP, Beckonert OP, Dumas ME (2011). A metabolic system-wide characterization of the pig: a model for human physiology.. Mol Biosyst.

[21] Iossa S, Lionetti L, Mollica MP, Crescenzo R, Botta M (2003). Effect of high-fat feeding on metabolic efficiency and mitochondrial oxidative capacity in adult rats.. Br J Nutr.

[22] Hoeks J, Briedé JJ, de Vogel J, Schaart G, Nabben M (2008). Mitochondrial function, content and ROS production in rat skeletal muscle: effect of high-fat feeding.. FEBS Lett.

[23] National Research Council (1996). Guide for the care and use of laboratory animals, 7^th^ ed.

[24] Liu JB, Chen DW, Mao XB, Yu B (2011). Effects of maternal folic acid supplementation on morphology and apoptosis-related gene expression in jejunum of newborn intrauterine growth retarded piglets.. Arch Anim Nutr.

[25] National Research Council (1998). Nutrient Requirements for Swine, 10th ed.

[26] He J, Chen DW, Yu B (2010). Metabolic and transcriptomic responses of weaned pigs induced by different dietary amylose and amylopectin ratio.. PLoS One.

[27] Ide T, Watanabe M, Sugano M, Yamamoto I (1987). Activities of liver mitochondrial and peroxisomal fatty acid oxidation enzymes in rats fed trans fat.. Lipids.

[28] Gardner PR (2002). Aconitase: sensitive target and measure of superoxide.. Methods Enzymol.

[29] Kouba M, Mourot J (1999). Effect of a high linoleic acid diet on lipogenic enzyme activities and on the composition of the lipid fraction of fat and lean tissues in the pig.. Meat Science.

[30] Barrientos A (2002). In vivo and in organello assessment of OXPHOS activities.. Methods.

[31] Serviddio G, Bellanti F, Romano AD, Tamborra R, Rollo T (2007). Bioenergetics in aging: mitochondrial proton leak in aging rat liver, kidney and heart.. Redox Rep.

[32] Pfaffl MW (2001). A new mathematical model for relative quantification in realtime RT-PCR.. Nucleic Acids Res.

[33] Rueda-Clausen CF, Dolinsky VW, Morton JS, Proctor SD, Dyck JR (2011). Hypoxia-induced intrauterine growth restriction increases the susceptibility of rats to high-fat diet-induced metabolic syndrome.. Diabetes.

[34] Lane RH, Kelley DE, Ritov VH, Tsirka AE, Gruetzmacher EM (2001). Altered expression and function of mitochondrial β-oxidation enzymes in juvenile intrauterine-growth-retarded rat skeletal muscle.. Pediatr Res.

[35] Taylor PD, Poston L (2007). Developmental programming of obesity in mammals.. Exp Physiol.

[36] Morise A, Sève B, Macé K, Magliola C, Le Huërou-Luron I (2011). Growth, body composition and hormonal status of growing pigs exhibiting a normal or small weight at birth and exposed to a neonatal diet enriched in proteins.. Br J Nutr.

[37] Poore KR, Fowden AL (2004). The effects of birth weight and postnatal growth patterns on fat depth and plasma leptin concentrations in juvenile and adult pigs.. J Physiol.

[38] Jacob S, Machann J, Rett K, Brechtel K, Volk A (1999). Association of increased intramyocellular lipid content with insulin resistance in lean nondiabetic offspring of type 2 diabetic subjects.. Diabetes.

[39] Boden G (1997). Role of fatty acids in the pathogenesis of insulin resistance and NIDDM.. Diabetes.

[40] Simmons RA (2007). Developmental origins of diabetes: the role of epigenetic mechanisms.. Curr Opin Endocrinol Diabetes Obes.

[41] Desai M, Gayle D, Babu J, Ross MG (2007). The timing of nutrient restriction during rat pregnancy/lactation alters metabolic syndrome phenotype.. Am J Obstet Gynecol.

[42] Coupé B, Grit I, Dominique D, Parnet P (2009). The timing of “catch-up growth” affects metabolism and appetite regulation in male rats born with intrauterine growth restriction.. Am J Physiol Regul Integr Comp Physiol.

[43] Gondreta F, Lefaucheura L, Louveaua I, Lebreta B, Pichodob X (2005). Influence of piglet birth weight on postnatal growth performance, tissue lipogenic capacity and muscle histological traits at market weight.. Livestock Production Science.

[44] Powell SE, Aberle ED (1980). Effects of birth weight on growth and carcass composition of swine.. J Anim Sci.

[45] Sreekumar R, Nair KS (2007). Skeletal muscle mitochondrial dysfunction & diabetes.. Indian J Med Res.

[46] Li ZY, Yang Y, Ming M, Liu B (2011). Mitochondrial ROS generation for regulation of autophagic pathways in cancer.. Biochem Biophys Res Commun.

[47] Ogata ES, Swanson SL, Collins JW, Finley SL (1990). Intrauterine growth retardation: altered hepatic energy and redox states in the fetal rats.. Pediatr Res.

[48] Serviddio G, Bellanti F, Giudetti AM, Gnoni GV, Petrella A (2010). A silybin-phospholipid complex prevents mitochondrial dysfunction in a rodent model of nonalcoholic steatohepatitis.. J Pharmacol Exp Ther.

[49] Lee HK, Song JH, Shin CS, Park DJ, Park KS (1998). Decreased mitochondrial DNA content in peripheral blood precedes the development of non-insulin-dependent diabetes mellitus.. Diabetes Res Clin Pract.

[50] Raffaella C, Francesca B, Italia F, Marina P, Giovanna L (2008). Alterations in hepatic mitochondrial compartment in a model of obesity and insulin resistance.. Obesity.

[51] Puigserver P, Spiegelman BM (2003). Peroxisome Proliferator-Activated Receptor-{gamma} Coactivator 1{alpha} (PGC-1{alpha}): Transcriptional Coactivator and Metabolic Regulator.. Endocr Rev.

[52] Ekstrand MI, Falkenberg M, Rantanen A, Park CB, Gaspari M (2004). Mitochondrial transcription factor A regulates mtDNA copy number in mammals.. Hum Mol Genet.

[53] Maniura-Weber K, Goffart S, Garstka HL, Montoya J, Wiesner RJ (2004). Transient overexpression of mitochondrial transcription factor A (TFAM) is sufficient to stimulate mitochondrial DNA transcription, but not sufficient to increase mtDNA copy number in cultured cells.. Nucleic Acids Res.

[54] Scarpulla RC (2006). Nuclear control of respiratory gene expression in mammalian cells.. J Cell Biochem.

[55] Jørgensen W, Gam C, Andersen JL, Schjerling P, Scheibye-Knudsen M (2009). Changed mitochondrial function by pre- and/or postpartum diet alterations in sheep.. Am J Physiol Endocrinol Metab.

[56] Petersen KF, Befroy D, Dufour S, Dziura J, Ariyan C (2003). Mitochondrial dysfunction in the elderly: possible role in insulin resistance.. Science.

[57] Lee YY, Lee HJ, Lee SS, Koh JS, Jin CJ (2011). Taurine supplementation restored the changes in pancreatic islet mitochondria in the fetal protein-malnourished rat.. Br J Nutr.

